# Collecting Social Media Information in a Substance Use Intervention Trial With Adolescent Girls With Lifetime Substance Use History: Observational Study

**DOI:** 10.2196/25405

**Published:** 2021-09-10

**Authors:** Lili M C Ramos, Joseline Delgadillo, Sarah Vélez, Emily Dauria, Jamie Salas, Marina Tolou-Shams

**Affiliations:** 1 Department of Psychology John Jay College of Criminal Justice and the Graduate Center City University of New York New York, NY United States; 2 Department of Psychiatry and Behavioral Sciences University of California, San Francisco San Francisco, CA United States

**Keywords:** adolescent girls, legal involvement, substance use, social media, health intervention

## Abstract

**Background:**

Adolescents with juvenile legal system contact face numerous barriers to participation in behavioral health intervention research, including housing disruption, legal privacy concerns, and systems mistrust. Technology, such as social media, may be a novel and developmentally appropriate adolescent research study engagement and retention tool.

**Objective:**

We examined data on social media information collected for study retention purposes from adolescents participating in a substance use intervention trial.

**Methods:**

Data were collected as part of a randomized controlled trial determining efficacy of a group-based substance use intervention for girls and young women (12-24 years) with substance use histories referred from legal and school systems in the United States. Baseline demographic and social media information was analyzed from the subset of 114 adolescent girls (mean age 15.7 years; range 13-18 years), of whom 31.6% (36/114) were legally involved, 87.7% (100/114) belonged to minoritized racial/ethnic groups, and 32.5% (37/114) received public assistance.

**Results:**

Most girls (74/114, 64.9%) provided at least one social media account (Instagram, 95% [70/74]; Facebook, 27% [20/74]; and Twitter, 11% [8/74]) during study enrollment. Legally involved girls were significantly less likely to provide social media information than school-referred girls (44% [16/36] versus 74% [58/78]; χ^2^_1_ [N=114]=9.68, *P*=.002).

**Conclusions:**

Obtaining social media information for study retention purposes from adolescent girls with lifetime substance use appears possible; however, particular subgroups (ie, legally involved girls) may be less likely to provide accounts. Factors shaping legally involved girls’ willingness to provide social media information, including mistrust and privacy concerns, and the impact of researcher’s access to social media information on study retention are critical directions for future research.

**Trial Registration:**

ClinicalTrials.gov NCT02293057; https://clinicaltrials.gov/ct2/show/NCT02293057

## Introduction

Large, representative samples in substance use intervention research are essential to best inform substance use treatment delivery policy and practices with adolescents with juvenile legal system contact (herein referred to as “legally involved adolescents”) and across the behavioral health cascade of care [1]. Prior research with legally involved adolescent populations has identified challenges to engaging and retaining participants in longitudinal intervention trials, suggesting a need for novel, age-specific contact methods [2,3]. High rates of social media use among adolescents in the United States are leading researchers to explore social media as a tool for recruiting, retaining, and intervening with adolescent research participants (eg, [4-6]), particularly those who may be harder to reach using traditional retention strategies (eg, in-person, voice calls). Social media allows adolescents to control how they present themselves to their social networks at a developmental stage when self-esteem, self-exploration, autonomy, and identity development matter significantly [7]. As adolescents rely increasingly on social media to fulfill these needs, their elevated time spent on the platforms suggests that social media could be a developmentally appropriate engagement strategy for researchers to reach and connect with adolescents on the platforms they already use to connect with peers, develop their individual identities, and carry out their day-to-day social lives [8].

Social media is also an efficient mechanism to recruit diverse (ie, in race/ethnicity, gender, socioeconomic status) adolescent groups into health research [6,9,10]. Social media may be especially useful for substance use intervention trials with legally involved adolescents, for whom researchers may face study recruitment and retention obstacles, such as disproportionate housing disruption that precludes consistent in-person contact [3,11] and high rates of household poverty that can result in frequent cellphone service disruption [12]. Social media platforms present a possible solution because they are accessible across multiple devices if phones are lost or stolen and via internet connections if phone plans are disrupted. Participants’ willingness to provide social media information for study communication must be examined, particularly among minoritized adolescent populations who may have differential willingness to provide social media information. Specifically, legally involved adolescents may be reluctant to provide social media information due to privacy concerns. Prior work has described how law enforcement agencies use social media for surveillance purposes, such as investigating and monitoring individuals’ activities online [13,14]. Adolescents who use substances may be especially reluctant to provide social media information due to concerns that images posted online might capture substance use and could lead to further system involvement. Surveillance by law enforcement has historically impacted People of Color disproportionately, which may also contribute to adolescents’ decisions to share social media information [13]. Empirical literature on using social media for study retention purposes to date has not included legally involved adolescent substance use research populations and has primarily focused on evaluating only 1 social media platform (ie, Facebook) [15,16].

As part of a larger substance use intervention trial with adolescent girls (legally involved and at-risk for legal involvement by virtue of substance use history), we examined data on social media account information provided to research staff for retention efforts. We hypothesized that legally involved girls would be less likely to provide social media information for study contact purposes than girls at-risk for legal involvement who were referred from schools (hereafter “school-referred girls”).

## Methods

This study includes baseline adolescent demographic and social media data collected between 2016 and 2019 as part of a federally funded, randomized controlled trial (Project VOICES) testing the efficacy of a gender-responsive, group-based substance use intervention [17] with girls and young women (12-24 years). To examine adolescent-specific patterns, we analyzed data from self-identified female adolescents (hereafter “girls”), aged 13-18 years (114/132, 86.4%) who could legally hold social media accounts according to the Children’s Online Privacy Protection Act (ie, age≥13 years). Eligible girls had to endorse lifetime history of substance use (alcohol, cannabis, or other drugs) on a private, computerized screener. Girls were ineligible for participation if their substance use treatment need required a higher level of care than an outpatient, if they chose to participate in an alternate substance use intervention, or had observable cognitive or developmental delays or active psychosis that would preclude group participation. Girls were recruited from juvenile probation and diversion and public school settings in a large metropolitan area on the West Coast of the United States. All girls referred from probation or diversion programs were living in the community (and not detained); school counselors referred girls from the schools who they determined might benefit from substance use intervention participation.

Eligible girls completed study assent, consent, and locator information for study follow-up. A Certificate of Confidentiality was obtained from the National Institutes of Health as an additional protection for participants’ privacy and was reviewed with adolescents prior to enrollment. For locator purposes, adolescents were asked to provide (1) cellphone numbers; (2) social media account information (Facebook, Instagram, or Twitter); (3) home address; and (4) contact information for at least three individuals who could help locate the adolescent if other contact methods were unsuccessful. Demographic data (eg, age, race, ethnicity) were collected as part of a private, computerized baseline assessment. Following the baseline assessment, girls were randomized to either an active (VOICES) or control (GIRLHealth; psychoeducational health curriculum) intervention, each consisting of twelve 60-minute group sessions (6-8 girls/group) (see [18] for a detailed description of full trial methods). Participants completed study assessments pre- (baseline)-, mid-, and immediate postintervention completion, and at 3 and 6 months after the intervention. All study procedures were approved by the Institutional Review Board of the Principal Investigator’s institution (MT-S; University of California, San Francisco).

## Results

Approximately one-third of girls were legally involved (36/114, 31.6%; Table 1). Girls were on average 16 years, predominantly belonged to minoritized racial and ethnic groups (12.3% [14/114] White, non-Hispanic/Latinx), and 32.5% (37/114) reported receipt of public assistance (eg, food stamps). There were no age (*t*_112_=0.847, *P*=.40) or race/ethnicity (χ^2^_4_ [*N=*114]=4.92, *P*=.30) differences between legally involved and school-referred girls. School-referred girls were less likely to report receipt of public assistance than were legally involved girls (χ^2^_1_ [*N=*114]=7.39, *P*=.007).

**Figure 1:**
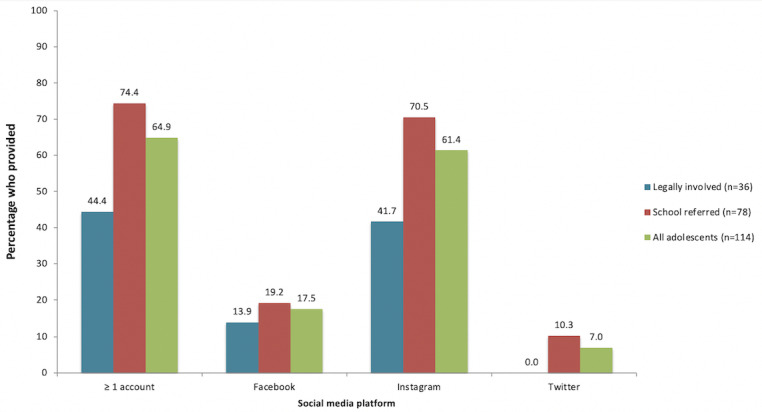
Social media account information provided by adolescent participants.

Most girls (74/114, 64.9%) provided at least one social media account as a study contact method (range 0-3; Figure 1); 70% (52/74) provided 1, 27% (20/74) provided 2, and 3% (2/74) provided 3. Of those, 95% (70/74) provided account information from Instagram, 27% (20/74) from Facebook, and 11% (8/74) from Twitter. Results from individual chi-square tests showed that girls were significantly more likely to provide an Instagram versus Twitter (χ^2^_1_ [*N=*114]=5.41, *P*=.02) account, with a similar pattern for Instagram versus Facebook (χ^2^_1_ [*N=*114]=3.54, *P*=.06). Girls’ provision of at least one social media account did not differ by race/ethnicity (χ^2^_4_ [*N=*114]=1.398, *P*=.85), socioeconomic status (χ^2^_1_ [*N=*114]=0.000, *P*=.99), or age (*t*_112_=–0.608, *P*=.54). Legally involved girls were significantly less likely to provide social media account information than school-referred girls (44% [16/36] versus 74% [58/78]; χ^2^_1_ [*N=*114]=9.68, *P*=.002).

**Table 1:** Demographic characteristics of adolescent participants (N=114).

Variable			Adolescents
Age at baseline (years), mean (SD)			15.7 (1.3)
**Race/ethnicity, *n* (%)**			
	White, non-Hispanic/Latinx	14 (12.3)	
	Black, African American or Haitian, non-Hispanic/Latinx	22 (19.3)	
	Mixed race or multiracial, non-Hispanic/Latinx	18 (15.8)	
	Other, non-Hispanic/Latinx^a^	15 (13.2)	
	Hispanic or Latinx	45 (39.5)	
**Legal system involvement, *n* (%)**			
	Legally involved	36 (31.6)	
	School referred	78 (68.4)	
**Self or family receiving public assistance, *n* (%)**			
	Yes	37 (32.5)	
	No	77 (67.5)	

^a^Includes participants who self-identified as Asian, Native Hawaiian or other Pacific Islander, American Indian, or “Other.”

## Discussion

The majority of school-referred and under half of legally involved adolescent girls (74% [58/78] and 44% [16/36], respectively) provided at least one social media account to researchers, suggesting that collecting social media information is possible among girls enrolled in substance use intervention trials. In comparison, national data on social media account ownership indicates that 72% of adolescents use Instagram and 51% use Facebook [19]. Social media ownership and utilization have not yet been examined among legally involved adolescents, yet adolescents belonging to minoritized racial and ethnic groups and adolescents with reported lower socioeconomic statuses, who are disproportionately represented in the juvenile legal system, demonstrate comparably high social media usage [12,19,20]. Further, adolescents who use substances are likely to use social media [21].

Consistent with our hypothesis, legally involved girls were significantly less likely than school-referred girls to provide social media information. Legally involved girls’ lesser willingness may be associated with disruptions in phone or device access (eg, due to caregivers or court taking cellphones away; lack of Wi-Fi) or concerns about privacy (eg, fears of court, probation or diversion staff, or parents monitoring) and system mistrust (eg, posting potentially self-incriminating images, researchers not keeping information from the court). Future research should examine how these and other factors may impact adolescents’ willingness to provide social media information, especially among legally involved girls. Differences in willingness to share social media information and limitations to using social media for research purposes with adolescents who may be most vulnerable to legal system monitoring should also be examined.

Our findings also suggest that girls might have preferred platforms for contact (ie, Instagram). This is in line with US adolescent trends from 2018 that Instagram use has surpassed Facebook in popularity [19]. It will be important in future research to assess whether the higher frequency of platforms provided (eg, Instagram in this study) is due to more prevalent account ownership or to greater willingness to release Instagram information to researchers relative to other platforms. To account for emergent social media trends potentially unknown to researchers (eg, TikTok), researchers should allow participants to provide open-ended responses when asked about social media use and contact preferences.

The use of an existing data set not designed for the purpose of these types of analyses comes with limitations. For example, this data set did not include reasons regarding willingness to provide social media information. Data were also limited to adolescent girls, which is informative for much needed gender-responsive substance use intervention trials, but our data do not address how to reach and engage legally involved adolescents who do not identify as female in substance use intervention research, nor the impact of gender identity on willingness to provide social media information. Girls also provided social media information in the presence of a consenting caregiver, which may have impacted their choices to share social media information. Future research should explore adolescents’ perspectives on the use of social media for study retention purposes, assess feasibility and acceptability using standardized measures, and qualitatively examine factors impacting decisions to share accounts (eg, privacy from parents, courts). Ethical implications of collecting social media information from adolescents for substance use intervention research retention purposes should also be considered in future research.

Our findings provide some of the first empirical data demonstrating that social media information can be collected to reach and retain historically minoritized, underrepresented, and vulnerable adolescent populations (ie, girls, legally involved, racial/ethnic minority) in substance use intervention research. Understanding more about willingness to provide account information and patterns of use, and assessing the effectiveness of social media use on improving study outreach, recruitment, and retention are critical areas for future research.
